# Plant protein-coding gene families: Their origin and evolution

**DOI:** 10.3389/fpls.2022.995746

**Published:** 2022-09-07

**Authors:** Yuanpeng Fang, Junmei Jiang, Xiaolong Hou, Jiyuan Guo, Xiangyang Li, Degang Zhao, Xin Xie

**Affiliations:** ^1^Key Laboratory of Agricultural Microbiology, College of Agriculture, Guizhou University, Guiyang, China; ^2^State Key Laboratory Breeding Base of Green Pesticide and Agricultural Bioengineering, Key Laboratory of Green Pesticide and Agricultural Bioengineering, Ministry of Education, Guizhou University, Guiyang, China; ^3^Department of Resources and Environment, Moutai Institute, Zunyi, China; ^4^Key Laboratory of Mountain Plant Resources Protection and Germplasm Innovation, Ministry of Education, College of Life Sciences, Institute of Agricultural Bioengineering, Guizhou University, Guiyang, China; ^5^Guizhou Conservation Technology Application Engineering Research Center, Guizhou Institute of Prataculture/Guizhou Institute of Biotechnology/Guizhou Academy of Agricultural Sciences, Guiyang, China

**Keywords:** plant evolution, gene families, molecular evolution, gene duplication, gene loss

## Abstract

Steady advances in genome sequencing methods have provided valuable insights into the evolutionary processes of several gene families in plants. At the core of plant biodiversity is an extensive genetic diversity with functional divergence and expansion of genes across gene families, representing unique phenomena. The evolution of gene families underpins the evolutionary history and development of plants and is the subject of this review. We discuss the implications of the molecular evolution of gene families in plants, as well as the potential contributions, challenges, and strategies associated with investigating phenotypic alterations to explain the origin of plants and their tolerance to environmental stresses.

## Introduction

The driving force underlying biological evolution is environmental selection. The criteria for plant diversification include marked interspecific phenotypic and genetic differences, which can be accompanied by marked reproductive isolation. However, by its very nature, plant evolution is a process wherein variations occur based on the presence, composition, and number of genes (Lafon-Placette et al., 2016). Interestingly, throughout this process, several important evolutionary mechanisms have dominated. These mechanisms include changes in drought resistance and oxygen uptake due to adaptation of plants to life on land (“landing”), formation of root and vascular structures, and evolution of metabolites in response to stress hazards. Additionally, co-evolution of floral structures has occurred in parallel with insects, leading to the co-evolution of insect mouthparts and floral diversity. Indeed, selected traits are often closely associated with the generation, development, and functional specialization of specific gene families (Gramzow et al., 2010; Cheng et al., 2019; Nikolov et al., 2019).

Horizontal gene transfer (HGT) may contribute to the adaptation of plants to life on land (Cheng et al., 2019), and has been documented in various gene families (Preston and Hileman, 2013; Shao et al., 2019). Moreover, several gene families are associated with repeated events, including tandem replication, fragment replication, wide-genome duplication (WGD), and transposable replication, leading to significant functional or phenotypic differences among plants (Wang et al., 2019, 2020; Schilling et al., 2020). For example, transposable replication often results in the formation of pseudogenes, while other types of replications cause a rapid expansion of plant genomes, leading to severe functional redundancy and increased functional differentiation in plant gene families. The presence of these redundant genes leads to a more complex adaptive system that drives plant-gene-phenotype-environment interactions, resulting in sub functionalization or *de novo* functionalization of these genes. This enables a coordinated and robust molecular network of environmental regulation in plants (Duplais et al., 2020; Man et al., 2020; Schilling et al., 2020).

A gene family is a group of genes with a common origin that encode proteins with similar structural properties and biochemical functions. Several key gene families, including *MADS* (Mcml Agamous Deficiens Srf-box domain gene family), *CYP* (Cytochrome P450 protein family), and *HSP* (Heat Shock Protein family), are core promoters of plant metabolism and flower formation (Ng and Yanofsky, 2001; Nelson and Werck-Reichhart, 2011; Bondino et al., 2012). For example, in the “ABCDE” model of flower development, the *MADS-box* genes are divided into two groups, namely, *M-type_MADS* and *MIKC_MADS*, with the latter considered to be the main contributor to flower development (Airoldi and Davies, 2012; Theissen et al., 2016; Hsu et al., 2021). In addition, evolutionary studies suggest extensive functional differentiation within these gene families and subfamilies. For example, the *CYP* gene family can be divided into two groups: type A-encoding genes, which encode oxygenases acting in pathways for the synthesis of plant-specific metabolites, including many chemosensory substances and drug components, and non-type A-encoding genes, which encode oxygenases required for the synthesis of more basic plant metabolites, such as endogenous plant hormones and essential metabolites (Ng and Yanofsky, 2001; Nelson and Werck-Reichhart, 2011; Airoldi and Davies, 2012; Theissen et al., 2016; Hsu et al., 2021; Su et al., 2021). Knowledge of the functional roles of plant gene families is vital to our understanding of plant evolution.

However, due to the richness of species and the associated wide range of gene families, the evolution of most gene families is poorly documented. This limits our in-depth exploration of plant origin and differentiation, as well as the application of molecular genetics. Therefore, evolutionary studies have taken a more comprehensive, multispecies approach.

## Plant evolution

**FIGURE 1:**
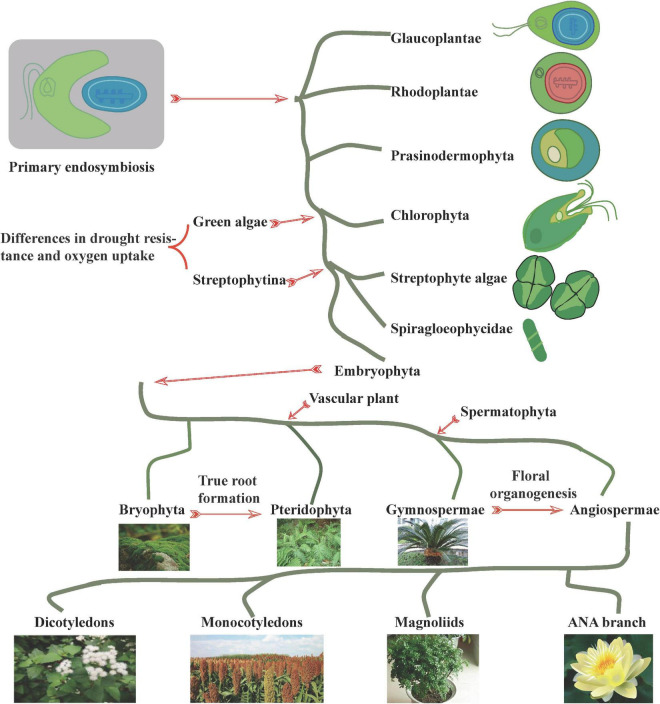
Plant evolution. The symbiosis of dinoflagellate protists with cyanobacteria prompted the occurrence of phytoplanktonic communities, with diverse phytoplanktonic taxa (including plants, green algae, red algae, and cryptophytes) arising through biological adaptation to the environment. At the origin of green algae and Streptophytina, significant differences in drought and oxygen stress tolerance developed to facilitate terrestrialization. During the process of adaptation to the environment, certain taxa underwent unique adaptations in root, flower, and other related phenotypes, which in turn ensured the dominance of the widely distributed angiosperms.

The evolution of plants from primitive plant ancestors has been largely simplified to red algae to green algae (basic green plants), mosses (basic land plants), ferns (basic vascular plants), gymnosperms (basic seed plants), and angiosperms. During this process, the phenotypes and genotypes of algae, mosses, ferns, and seed plants varied considerably. At the phenotypic level, selection of characteristics, such as plant type, leaf shape, and floral organs, is influenced by animal behavior, human activities, as well as climatic factors, leading to broad phenotypic diversity (Figure 1). At the genotypic level, abundant genetic changes such as WGD, tandem repeats, transposition, gene loss, and parallel gene transfer contribute significantly to the diversity of protein-coding plant genes and selective responses to the environment (Gramzow et al., 2010; Preston and Hileman, 2013; Cheng et al., 2019; Nikolov et al., 2019; Shao et al., 2019; Schilling et al., 2020).

Although the origin of terrestrial plants remains controversial, Cheng et al. (2019) reported that land plants might have originated from two Zygnematophyceae species, namely, *Spirogloea muscicola* and *Mesotaenium endlicherianum*. Cheng et al. (2019) and Liang et al. (2020) further reported that two species from outside the Streptophytina*—Mesostigma viride* and *Chlorokybus atmophyticus—*may represent the most primitive branches of terrestrialized plants. Further, genomic analysis identified Prasinodermaphyta as a potential new phylum between the green and red algal phyla (Li et al., 2020). Meanwhile, molecular analyses have revealed that mosses originated approximately 908–680 million years ago (Mya), suggesting that the origin of land plants occurred earlier than the Ordovician (Sun et al., 2021). Additionally, comparison of the genomes of magnolias indicates that Magnoliids and monocotyledons form a unique monophyletic group that may appear earlier than either the monocotyledon or the Austrobaileyales, Nymphaeales, and Amborellales (ANA) branches (Dong et al., 2021).

Based on genomic and transcriptomic analysis of representative bryophytes (including liverworts, hornworts, and mosses), Gao et al. (2020) noted that polyploidy was common in bryophytes. Polyploidization events occurred in bryophyte ancestors before differentiation, as well as within Funarioideae ancestors, and Buxbaumiidae, Diphysciidae, Timmiidae, and Funariidae branches. Schneider et al. (2017) found that polyploidization plays an important role in fern diversity. In fact, several instances of polyploidization contributed to the diversity of Asplenium plants, with ploidy levels of 2* and 4* being the most common. Meanwhile, two of the oldest polyploidization events were reported in seed plants (192 Mya) and angiosperms (319 Mya), during which genome multiplication was a hallmark of the evolution of angiosperms from gymnosperms (Schneider et al., 2017). In basal angiosperms, the ANA branch of camphor and water lily genomes indicates a polyploidization event in the water lily ancestor (Zhang et al., 2019). Similarly, magnolia genomes indicate that one polyploidization event occurred during their ancestry, while two additional polyploidization events occurred in Lauraceae. Wang et al. (2019) and Zhang L. S. et al. (2020) systematically organized the abundant polyploidy of angiosperms and confirmed that monocotyledonous plants from the Gramineae (100–110 Mya) and Lemnaceae (115–125 Mya) families are highly polyploid. Specifically, the orders Poales and Arecales appear to have had one polyploidization event, whereas plantains arose from three polyploidization events over a short period. Indeed, dicotyledonous plants are usually paleohexaploid (gamma triplication; 115–130 Mya), including Malvaceae, Brassicaceae, Cucurbitaceae, and Leguminosae, all of which originated following multiple ploidy events (Wang et al., 2019). Importantly, abundant gene duplications have also been reported in the genomes of other angiosperms, including sugarcane, kiwifruit, and tea tree (Vilela et al., 2017; Wang et al., 2018).

## Overview of plant gene families

A plant gene family refers to a group of genes with related functions that are generated by gene duplication from a single-copy gene source in an ancestor, and retain similar sequence and structure (Li et al., 2022). Gene families can be associated with repeated events, such as tandem replication, fragment replication, WGD, or transposable replication, based on the scope of replication, size of the replicated region, and influence of transposons (Airoldi and Davies, 2012; Su et al., 2021). Transposable replication is one such event that often leads to formation of pseudogenes, while other types of replications cause a rapid expansion of plant genomes, leading to severe functional redundancy and increased functional differentiation within plant gene families (Schilling et al., 2020; Yu et al., 2020).

**FIGURE 2:**
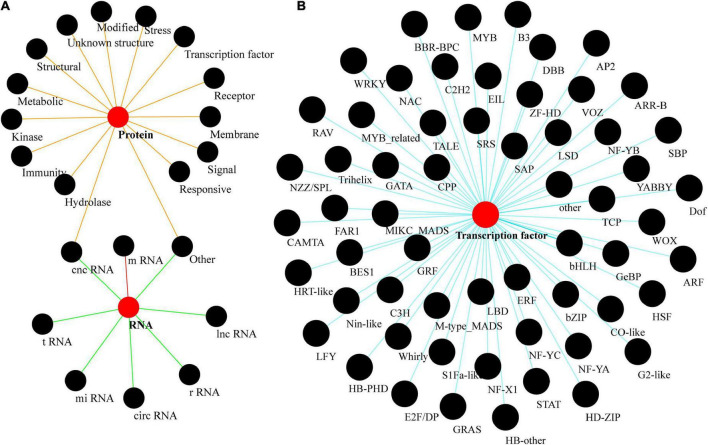
Plant gene families. **(A)** A brief classification of gene families found in plants. **(B)** A rich taxonomy of plant transcription factors. tRNA is an RNA composed of 76–90 nucleotides that carry amino acids into the ribosome and synthesize proteins under the guidance of mRNA; lnc RNA is a class of non-coding RNA molecules longer than 200 nt; mi RNA is a class of endogenous, small RNAs of about 20–24 nucleotides in length; circ RNA is a class of RNAs that do not have a 5′ terminal cap and 3′ terminal poly(A) tail, and are covalently bonded to form a loop structure; they are a class of non-coding RNA molecules that are found in living organisms. cnc RNA (coding and non-coding RNA) is a family of functional genes that can be differentially sheared in a variable manner, resulting in both short peptides or small molecular weight proteins and untranslatable functional RNAs (e.g., Inc RNA, mi RNA, etc.).

Plant genomes include protein-coding and non-coding RNA (ncRNA) gene families (Song et al., 2021; Li et al., 2022). Gene families encoding ncRNA can be further subdivided into those encoding lncRNA (long non-coding RNA), miRNA (micro RNA), rRNA (ribosomal RNA), tRNA (transfer RNA), and circRNA (circular RNA), and will not be further discussed here. Protein-coding gene families can also be broadly classified by the function of the proteins they encode, including receptors, kinases, epigenetic modification, structural, and transcription factors (TFs) (Figure 2A). However, these classifications are not unique; gene families can also be divided into several categories depending upon the classification criteria, such as classifications based on function, structural features, or the pathways involved. Hence, the class of chloroplast transporters TOC-TIC can be classified as either membrane proteins or structural proteins, whereas G-protein-coupled signal receptors can be classified as either membranes or receptor proteins. Many gene families within plant genomes are unique to plants, including more than 57 families of TFs, e.g., the TEOSINTE BRANCHED 1/CYCLOIDEA/PROLIFERATING CELL FACTOR (*TCP*), and SQUAMOSA PROMOTER-BINDING PROTEIN (*SBP*) families (Figure 2B; Reeves and Olmstead, 2003; Yang et al., 2008; Preston and Hileman, 2013; Jin et al., 2017; Wu et al., 2017).

## Evolution of gene families in plants

### Evolution of resistance gene families

**FIGURE 3:**
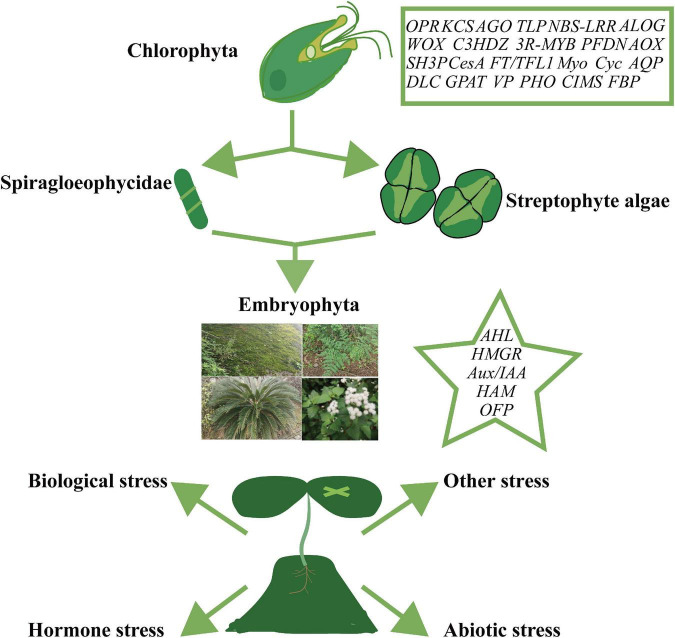
Origin and expansion of plant gene families. Gene families in boxes representing their origin in green algae or earlier. Families include OPR (12-oxo-phytodienoate acid reductase), KCS (3-ketoacyl-coa synthase), AGO (Argonaute), TLP (thaumatin-like protein), NBS-LRR (nucleotide binding site leucine-rich repeat), ALOG (Arabidopsis LSH1 and Oryza G1), WOX (WUSCHEL-related), C3HDZ (class III homeodomain-zinc finger protein), 3R-MYB, PFDN (prefoldin), AOX (alternative oxidase), SH3P (SH3 and BAR domain-containing protein), CesA (cellulose synthase), FT/TFL1 (flowering locus t/terminal flower 1), Myo (myosin), Cyc (cyclin), AQP (aquaporins), DLC (dynein light chain), GPAT (glycerol-3-phosphate acyltransferase), VP (vacuolar-type H+-pyrophosphatase), PHO (phosphate 1), CIMS (cobalamin-independent methionine synthase), and FBP (F-box). Gene families listed in the star may have contributed to the development of Streptophyte algae or functional innovations in the plant community, and include AHL (AT-hook motif nuclear localized), HMGR (3-hydroxy-3-methylglutaryl coenzyme A reductase), Aux/IAA (auxin/indole acetic acid and auxin response factor), HAM (hairy meristem), and OFP (OVATE family protein).

Resistance genes are groups of genes encoding proteins required for tolerance or immunity during plant adaptation to adverse external stress. Multiple environmental stresses have driven the molecular selection of these genes. Resistance gene clusters such as the *NBS-LRR* family are large and exhibit a high degree of functional differentiation (Shao et al., 2019). *HSP* and *sHSP* encode important heat-responsive proteins and molecular chaperones, and the copy number of *sHSPs* is significantly increased in polyploid plants with multiple branches. Genes from different subclasses may have diversified in function (Bondino et al., 2012). In contrast, the molecular chaperone gene *PFDN*, which displays only marginal differences between different groups, is expanded in polyploid plants such as soybean (Cao et al., 2016). Furthermore, the number of chilling injury-related gene (*CRG*) family members in Cruciferae is affected by polyploidy (Song et al., 2020). On the other hand, evolution of the *AOX* gene family is primarily mediated by intron/exon loss or gain, and fragment deletion, although gene loss and duplication, as well as tandem blocking, also play essential roles in the origin and maintenance of the family (Pu et al., 2015; Tables 1, 2; Figure 3).

Natural selection often drives the evolution of disease resistance-related genes to establish functional differentiation between these genes, with various external hazards leading to the vast expansion of the genes. For example, there are many structural variations in the leucine-rich repeat receptor-like kinase (*LRR-RLK)* gene family (Man et al., 2020). The resistance I genes from the *NBS-LRR* superfamily originated from Chlorophyta (green algae) and were classified into five categories according to their structural characteristics [Chlorophyta: RNL; Charophyta: CNL; Embryophyta (land plants): TNL, HNL, and PNL] (Shao et al., 2019). *NLR* genes (*CNL*, *TNL*) are clearly classified as being found in Solanaceae species; however, their prevalence varies markedly, with few reported within the genome of tomato plants and many more in those of potatoes and peppers (Borrelli et al., 2018). Another example is offered by the evolution of the *AGO* gene family, which encodes proteins associated with antiviral activity. This family may have experienced 133–143 repeat events and 272–299 loss events, including five major repeats. Specifically, the differentiation of green algae may have formed four major branches (I: 1/10, II: 5, III: 4/6/8/9, IV: 2/3/7) of the *AGO* gene family (Singh et al., 2015). Similarly, the *DRB* gene family is divided into two branches based on differences in the number of double-stranded RNA binding motifs (dsRBM); the number of DRB proteins also varies among different species (Clavel et al., 2016). The plant *RDR* (RNA-dependent RNA enzyme) family originated from copies of three monophyletic genes, *RDR*α, *RDR*β, and *RDR*γ, and was dependent on species divergence (Zong et al., 2009). Plant *DCL* (Dicer-like), however, followed the evolutionary traces of early plant evolution through independent replication, remodeling its RNA binding pocket in response to virus resistance (Mukherjee et al., 2013). Finally, expansion of the *TLP* gene family in green algae (1), mosses (6), and angiosperms (>20), may be based on tandem and segmental duplication events (Cao et al., 2016; Tables 1, 2; Figure 3).

**TABLE 1:** Structural analysis of plant protein-coding gene families.

Gene family	Abbreviation	Major function	Domain	References
**Metabolic enzymes**				
Cytochrome P450	*CYP/P450*	Monooxygenation activity	P450	Su et al., 2021
12-oxo-phytodienoate acid reductase	*OPR*	Jasmonic acid biosynthesis	Unknown	Guo et al., 2016
3-hydroxy-3-methylglutaryl Coenzyme A Reductase	*HMGR*	Terpene synthesis	PF00368	Li et al., 2014
Aconitase	*ACO*	Catalyzes the Isomerization of citrate to isocitrate	ACO	Wang et al., 2016
3-ketoacyl-coa synthase	*KCS*	Very long-chain fatty acids (VLCFAS) synthesis	ACP synthase III C and like	Guo et al., 2016
**Antiviral gene cluster**				
Leucine-rich repeats Receptor-like protein kinases	LRR-RLK	Perceptual signaling and phosphorylation	LRR and RLK	Man et al., 2020
Argonaute	*AGO*	Antiviral activity	PAZ and Piwi	Singh et al., 2015
Double stranded RNA binding protein	*DRB*	Antiviral activity	DSRM	Clavel et al., 2016
Thaumatin-like protein	*TLP*	Plant disease resistance	TLP	Cao et al., 2016
Nucleotide-binding leucine-rich repeat	*NLR*	Plant disease resistance	NB-ARC	Borrelli et al., 2018
Nucleotide binding site leucine-rich repeat	*NBS-LRR*	Plant disease resistance	LRR and NBS	Shao et al., 2019
**Transcription factor cluster**				
\	*MADS*	Flower development	MADS	Gramzow et al., 2010
AT-hook Motif Nuclear Localized	*AHL*	Organ development and bulky	AT-hook and PPC	Zhao et al., 2014
Arabidopsis LSH1 and Oryza G1	*ALOG*	Regulate reproductive growth	Unknown	Naramoto et al., 2020
Auxin/Indole Acetic Acid and Auxin Response Factor	*Aux/IAA*	Auxin response	Aux/IAA	Wu et al., 2017
Cysteine-rich polycomb-like protein	*CPP-like*	Development of reproductive organs	CXC	Yang et al., 2008
Wuschel-related	*WOX*	Regulating cell division and differentiation	WOX	Lian et al., 2014
Class III Homeodomain-Zine finger protein	*C3HDZ*	Leaf growth	HD-ZIP	Vasco et al., 2016
\	*YABBY*	Leaf growth	YABBY	Finet et al., 2016
\	*3R-MYB*	Drought and development	3 MYB	Feng et al., 2017
**Anti-stress gene cluster**				
Small heat shock protein/alpha-crystallin	*sHSP/Cry*	Molecular chaperone	HSP20	Bondino et al., 2012
Prefoldin	*PFDN*	Molecular chaperone	Prefoldin	Cao, 2016
Cold-related genes	*CRG*	Cold-related	Unknown	Song et al., 2020
Alternative oxidase	*AOX*	Ubiquinol to reduce oxygen to water	Unknown	Pu et al., 2015
**Structural composition or organogenesis gene cluster**				
SH3 and BAR domain-containing protein	*SH3P*	The Plant Cell Division and Autophagy	BAR domain	Forero and Cvrckova, 2019
Hairy meristem	*HAM*	Meristem formation	GRAS	Geng et al., 2021
Cellulose synthase	*CesA*	Cellulose synthesis	Cellulose_synt, Glycos_transf_2 and Glyco_trans_2_3	Little et al., 2018
Flowering locus t/terminal flower 1	*FT/TFLl*	Flower development	Unknown	Jin et al., 2021
Myosin	*Myo*	Actin system	Unknown	Peremyslov et al., 2011
Alternative splicing modulators nuclear speckle rna-binding proteins	*NSR/RBP*	Gene expression	Unknown	Lucero et al., 2020
Cyclin	*Cyc*	Cycle control	Cyclin_N and Cyclin_C	Boscolo-Galazzo et al., 2021
OVATE family protein	*OFP*	Fruit shape regulation	OVATE	Liu et al., 2014
Aquaporins	*AQP*	Water inflow and cycle control	Unknown	Hussain et al., 2020
Dynein light chain	*DLC*	Dynein complexes	4 helix and 4 sheet	Cao et al., 2017
Psbp protein	*PsbP*	Oxygen-evolving complex (OEC)	I and II	Ifuku et al., 2008
**Signal-mediated gene clusters**				
Calcineurin B-Like and CBL-Interacting Protein Kinase	*CBL/CIPK*	Ca^2+^ signal	CBL/CIPK/C2	Zhang X. X. et al., 2020
Calcium-dependent protein kinase and CDPK-related kinase	*CDPK/CRK*	Ca^2+^ signal	CDPK/CRK/C2	Xiao et al., 2017
Glycerol-3-phosphate acyltransferase	*GPAT*	Phospholipid signal	acyltransferase	Waschburger et al., 2018
Phosphatidyl ethanolamine binding protein	*PEBP/MFT-like*	Phospholipid signal	Unknown	Hedman et al., 2009; Karlgren et al., 2011
Rapid alkalization factor	*RALF*	PH rise induction	Unknown	Cao and Shi, 2012
Auxin response factor	*ARF*	Auxin signal transduction	ARF	Finet et al., 2013
Cyclic nucleotide-gated ion channel	*CNGC*	Calcium signal transduction	CNB	Saand et al., 2015
C-terminally encoded peptide	*CEP*	Small secreted peptide signals	CEP	Ogilvie et al., 2014
Poly(A)-binding protein	*PAB*	Promoting mrna integrity and protein synthesis	PABP	Gallie and Liu, 2014
**Supply of nutrients or ions gene clusters**				
Vacuolar iron transporter	*VIT*	Iron sensing and transport	VIT	Cao, 2019
Ferritin	*Fer*	Iron sensing and transport	Unknown	Strozycki et al., 2010
H+-ppase	*VP*	Proton-translocating pyrophosphatase	TM1-16	Zhang Y. M. et al., 2020
Phosphate 1	*PHO*	Inorganic phosphate (Pi) sensing and transport	SPX, EXS	He et al., 2013
Cobalamin-independent methionine synthase	*CIMS*	Cobalamin-independent methionine synthase	Unknown	Rody and de Oliveira, 2018
**Hydrolase gene clusters**				
B -amylase	*BAM*	Glucan hydrolytic	Unknown	Thalmann et al., 2019
Sucrose synthase	*SUS*	Sugar hydrolysis	Unknown	Xu et al., 2019
**Apparent components gene clusters**				
Histone methyltransferases	*HMT*	Methylation process	Unknown	Zhao et al., 2018
F-box	*FBP*	Ubiquitylation process	F-box	Navarro-Quezada et al., 2013

Major function indicates the most important functional role of gene families; domain refers to a conserved region of a protein sequence that may be related to the functional site of the protein. Some gene families are marked with a domain labeled “Unknown” to denote that a specific model of their overall structure is not currently known, and the methods available for further discovery of new sequences can only rely on the appropriate “blast p” homology search. For such proteins, a larger scale phylogenetic exploration may be useful to infer and resolve their function and structure.

**TABLE 2:** Evolutionary events of plant protein-coding gene families.

Gene family	Numbers	Coverage	Copy event	Contribution to genome-wide repeating events	Stage of event	References
**Metabolic enzymes**						
*CYP/P450*	251	Unknown	Order level and below level	B_1_	Unknown	Su et al., 2021
*OPR*	6	A_1_, 11	Order level and below level	B_1_	Chlorophyta, unknown	Li et al., 2009
*HMGR*	2	A_1_, 20	Species level	B_1_	Moss, unknown	Li et al., 2014
*ACO*	3	A_2_, 12	Species level	B_1_	Unknown	Wang et al., 2016
*KCS*	11	A_1_, 28	Order level and below level	B_1_	Chlorophyta, unknown	Li et al., 2009
**Antiviral**						
*LRR-RLK*	225	A_2_, 9	Species level	B_1_	Unknown	Man et al., 2020
*AGO*	10	A_1_, 30	Order level and below level	B_1_	Chlorophyta, unknown	Singh et al., 2015
*DRB*	7	A_5_, 15	Species level	B_1_	Unknown	Clavel et al., 2016
*TLP*	24	A_1_, 6	Order level and below level	B_1_	Chlorophyta, unknown	Cao et al., 2016
*NLR*	144	A_5_, 3	Species level	B_1_	Unknown	Borrelli et al., 2018
*NBS-LRR*	204	A_0_, 79	Order level and below level	B_1_	Chlorophyta, unknown	Shao et al., 2019
**Transcription factors**						
*MADS*	43	A_0_, Unknown	Order level and below level	B_1_	Earlier, MRCA	Gramzow et al., 2010
*AHL*	29	A_1_, 19	Order level and below level	B_1_	Moss, unknown	Zhao et al., 2014
*ALOG*	10	A_1_, 9	Order level and below level	B_1_	Chlorophyta, ALOS1	Naramoto et al., 2020
*Aux/IAA*	29	A_1_, 17	Order level and below level	B_1_	Moss, unknown	Wu et al., 2017
*CPP-like*	8	A_4_, 2	Unknown	B_0_	Unknown	Yang et al., 2008
*WOX*	16	A_0_, 50	Order level and below level	B_1_	Chlorophyta, unknown	Lian et al., 2014
*C3HDZ*	5	A_1_, 32	Order level and below level	B_1_	Chlorophyta, unknown	Vasco et al., 2016
*YABBY*	6	A_3_, 50	Species level	B_1_	Unknown	Finet et al., 2016
*3R-MYB*	5	A_1_, 65	Order level and below level	B_1_	Chlorophyta, unknown	Feng et al., 2017
**Anti-stress**						
*sHSP/Cry*	27	A_4_, 17	Species level	B_1_	Unknown	Bondino et al., 2012
*PFDN*	9	A_1_, 14	Family level	B1	Chlorophyta, unknown	Cao et al., 2016
*CRG*	420	A_2_, 21	Species level	B1	Unknown	Song et al., 2020
*AOX*	5	A_1_, Unknown	Order level and below level	B_1_	Charophyta, *AOX1* and *AOX2*	Pu et al., 2015
**Structural composition or organogenesis**						
*SH3P*	3	A_1_, 20	Family level of angiosperms	B_0_	Charophyta, *SH3P1*	Lucero et al., 2020
*HAM*	3	A_1_, 42	Order level and below level	B_1_	Moss, unknown	Liu et al., 2014
*CesA*	26	A_4_, 46	Order level and below level	B_1_	Charophyta, unknown	Guo et al., 2016
*FT/TFLl*	6	A_1_, Unknown	Order level and below level	B_1_	Charophyta, MFT-like	Forero and Cvrckova, 2019
*Myo*	17	A_1_, 12	Order level and below level	B_0_	Charophyta, *myo-xi (a)*	Cao et al., 2017
*NSR/RBP*	2	A_5_, 7	Species level	B_1_	Unknown	Hussain et al., 2020
*Cyc*	50	A_1_, 10	Order level and below level	B_1_	Chlorophyta, unknown	Ifuku et al., 2008
*OFP*	19	A_1_, 19	Species level	B_1_	Moss, unknown	Jin et al., 2021
*AQP*	35	A_1_, 24	Order level and below level	B_1_	Chlorophyta, *lips*	Peremyslov et al., 2011
*DLC*	6	A_1_, 15	Order level and below level	B_1_	Chlorophyta, *DLC-VIII*	Boscolo-Galazzo et al., 2021
*PsbP*	2	Unknown	Unknown	B_0_	Unknown	Little et al., 2018
**Signal transduction**						
*CBL/CIPK*	14/35	A_2_, 18	Order level and below level	B_1_	Unknown	Xiao et al., 2017
*CDPK/CRK*	34/8	A_3_, 6	Family level	B_1_	Unknown	Cao and Shi, 2012
*GPAT*	10	A_1_, 39	Order level and below level	B_1_	Chlorophyta, *GPAT* and *GPAT9*	Karlgren et al., 2011
*PEBP*	6	A_3_, 106	Order level and below level	B_1_	Unknown	Hedman et al., 2009; Zhang X. X. et al., 2020
*RALF*	33	A_4_, 4	Family level	B_1_	Unknown	Finet et al., 2013
*ARF*	23	A_2_, 21	Unknown	B_0_	Unknown	Saand et al., 2015
*CNGC*	20	A_4_, 15	Unknown	B_0_	Unknown	Ogilvie et al., 2014
*CEP*	12	A_3_, 106	Order level and below level	B_1_	Unknown	Gallie and Liu, 2014
*PAB*	8	A_1_, 54	Unknown	B_1_	Unknown	Geng et al., 2021
**Supply of nutrients or ions**						
*VIT*	6	A_1_, 14	Angiosperms	B_0_	Unknown	Strozycki et al., 2010
*Fer*	4	A_0_, 16	Order level and below level	B_0_	Unknown	Zhang Y. M. et al., 2020
*VP*	3	A_0_, 27	Order level and below level	B_1_	Rhodoplantae and Chlorophyta, unknown	He et al., 2013
*PHO*	9	A_1_, 32	Order level and below level	B_1_	Chlorophyta, unknown	Geng et al., 2021
*CIMS*	3	A_1_, 35	Species level	B_1_	Chlorophyta, unknown	Cao, 2019
**Hydrolases**						
*BAM*	10	A_0_, 136	Order level and below level	B_1_	Unknown	Rody and de Oliveira, 2018
*SUS*	6	A_4_, 16	Species level	B_1_	Unknown	Thalmann et al., 2019
**Other components**						
*HMT*	3	A_2_, 29	Unknown	B_0_	Unknown	Xu et al., 2019
*FBP*	211	A_1_, 34	Order level and below level	B_1_	Chlorophyta, unknown	Zhao et al., 2018

A0, Archaeplastida populations; A1, green plant population; A2, land plant population; A3, seed plant population; A4, angiosperm population; A5, dicotyledonous plant population. For the contributions made to the genome-wide repeat events (such as paleopolyploidization and WGD), B0 indicates that no effect was observed or had been studied, and BN indicates an effect caused by N repeats. The copy event refers to the level of replication events that impact copy number.

### Evolution of transcription factor gene families

Transcription factors function as regulatory elements of various plant processes, including growth, the stress response, and reproduction (Yang et al., 2008; Lian et al., 2014; Zhao et al., 2014; Finet et al., 2016; Vasco et al., 2016; Feng et al., 2017; Wu et al., 2017; Naramoto et al., 2020). Due to the rich evolutionary history of plants, TF gene families tend to have more members and a higher degree of functional differentiation compared with structural protein-related coding genes (Finet et al., 2016). In particular, the *AHL* gene family, which is related to plant growth and development, may have evolved from the fusion of algal PPC structural proteins and AT-hook motifs, and is thought to have originated in bryophytes. This family can be divided into three groups (A: I; B: II, III), with a high degree of gene loss and numerous duplication events throughout evolution (Zhao et al., 2014). The *WOX* gene family, which is involved in cell division, originated in green algae and is primarily divided into nine classes (WOX1/2, WOX5/7, WOX3, WOX4, WOX6, WOX11/12, WOX13, and WUS) with WOX13 being recognized as the oldest branch. Indeed, WOX genes exhibit significant variation in their motifs and number of members throughout their evolutionary process (Lian et al., 2014). CPP-like genes, which are associated with plant development, are divided into four branches: Gene deletion and species-specific amplification have been important in expanding this gene family, while positive selection has served as the primary evolutionary driving force (Yang et al., 2008).

The *SPL/SBP* family mainly includes nine subbranches, among which there are obvious evolutionary differences; their formation may be completed before the differentiation of the angiosperms (Preston and Hileman, 2013). The nine evolutionary branches, namely, *SPL* evolutionary branch-I, evolutionary branch-II, evolutionary branch-IV, evolutionary branch-V, evolutionary branch-VI, evolutionary branch-VII, evolutionary branch-VIII, and evolutionary branch-IX, are characterized by differences in function and altered mi RNA regulatory differences (Preston and Hileman, 2013). The *TCP* gene family consists of two main classes (classes I and II, i.e.: the CIN and CYC/TB1 evolutionary branches) (Liu et al., 2019). Among them, all land plants have CIN evolutionary branch *TCP* genes, while CYC evolutionary branch genes are only found in true dicotyledons and monocotyledons (Liu et al., 2019). In addition, the rapid expansion of the *TCP* gene family is consistent with a polyploidy trend in land plants, with fewer tandem duplication events (Liu et al., 2019). *3R-MYB* is a regulatory TF associated with drought-resistance and development. Its structure is progressively more complex in different species groups, in conjunction with a gradual increase in the number of gene family members, forming three branches (A, B, and C3) in angiosperms (Feng et al., 2017). The family of *ALOG* genes, which regulate reproductive growth, originated in green algae and expanded significantly in angiosperms (Naramoto et al., 2020). The *YABBY* and *C3HDZ* gene families, associated with leaf growth, have evolved in stages of biological evolution and their molecular structures have given rise to several major branches with different molecular classes exerting unique effects on leaf development (Finet et al., 2016; Vasco et al., 2016).

Moreover, the *MADS* and *AUX/IAA* gene families originated in early land plants (mosses) and expanded to encompass multiple gene sub-family classes that have shown rich functional differentiation with multiple rounds of evolutionary events (Theissen et al., 2016; Wu et al., 2017). Specifically, the MADS domains in plants originated from the transformation of topoisomerase IIA subunit A (*TOPOIIA-A*) into *MRCA* and the latter’s subsequent modification to SRF-like and MEF2-like MADS-box genes. Furthermore, in angiosperms, type II *MADS-box* genes mediate major evolutionary innovations in plant flowers, ovules and fruits, whereas the formation of the *M*γ and interacting *M*α genes (*M*α*) of type I *MADS-box* can be traced back to the angiosperm ancestor and may be related to its heterodimeric function in angiosperm-specific embryonic trophoblast endosperm tissue (Qiu and Claudia, 2021). This evolutionary process was affected by various events, including replication and functional differentiation, resulting in the functional diversity of their regulatory properties (Ng and Yanofsky, 2001; Gramzow et al., 2010; Airoldi and Davies, 2012; Theissen et al., 2016; Schilling et al., 2020; Hsu et al., 2021; Tables 1, 2; Figure 3).

### Evolution of metabolic enzyme gene families

Metabolites are a direct manifestation of plant physiology. Highly specific biochemical processes that produce various metabolites have driven the formation and functional specialization of metabolic gene clusters (Duplais et al., 2020). Studies investigating the recurring events that led to the development of plant metabolic enzyme gene clusters have revealed a close relationship among the different metabolites (Duplais et al., 2020). The *CYP*/*P450* gene family of mono-oxygenases is highly abundant in angiosperms, possibly due to multiple repeated events (polyploidy, tandem replication, and fragment repeat). They can be divided into two categories, A-type (e.g., CYP71) and non-A-type (e.g., CYP51, CYP72, CYP74, CYP85, CYP86, CYP97, CYP710, CYP711, CYP727, and CYP746), with CYP51 and CYP97 potentially representing the oldest clades (Su et al., 2021). The *ACO* gene families associated with respiration were almost lost early in the evolutionary path; however, they subsequently expanded and currently exist as large, functionally distinct subclasses (Wang et al., 2016; Tables 1, 2; Figure 3).

The *OPR* gene family of jasmonic acid biosynthesis-related enzymes doubled in number during the evolution of algae to land plants and further expanded *via* polyploidization and tandem duplication events. This gene family comprises seven categories. All *OPR* genes from green algae form subclade VII, subclade VI (present only in lower land plants), and subclade II (present in all land plants except the gymnosperm *Picea sitchensis*); subclade I is composed of gymnosperm and angiosperm sequences. Only monocotyledon sequences comprise subbranches III, IV, and V. The *OPR* gene family is particularly abundant in rice and sorghum (13 genes) (Li et al., 2009).

The *HMGR* gene family is associated with terpene biosynthesis and originated from bryophytes. It has only expanded in maize, soybean, cotton, and poplar, with each species containing five *HMGR* genes (sporophyte-specific branch, monocotyledon-specific branch HMGR III/IV, and dicotyledon-specific branch HMGR I/II) with different conserved sequences (Li et al., 2014).

The *KCS* gene family, which is involved in ultra-long-chain fatty acid synthesis, is divided into five main sub-clades (A, B, C, D, and E) with the number of genes in this family gradually increasing from one in algae to eleven in angiosperms, and with an apparent trend in the expansion of related polyploid species (Little et al., 2018).

### Evolution of protein families associated with plant cell structure

Proteins with roles in cell wall formation and other aspects of cell structure are important for plant morphogenesis and can have basic enzymatic reactions. These proteins tend to have a low probability of gene loss, but they can accumulate a high degree of functional differentiation throughout a long evolutionary process, as observed within the *CesA* family of cellulose synthases (Little et al., 2018). The *PSBP* gene, encoding the light-harvesting protein complex PSII, only exists in the green plants of polymorphic biological groups that consist of few members with obvious structural differences (Ifuku et al., 2008). Cell cycle-related *Cyc* genes are divided into ten branches, most of which existed before green algae and became widely expanded during the transition to angiosperms (Boscolo-Galazzo et al., 2021). *DLC* genes associated with the dynein system are derived from *DLC*-VIII genes of green algae. With the gradual expansion of *DLC* genes along the evolutionary path, each plant type produced unique molecules (e.g., algae: DLC-VIII, bryophyte: DLC-VII, fern: DLC-IV, monocotyledon: DLC-I/II, dicotyledon: II/V), with a common branch in seed plants (DLC-VI) (Cao et al., 2017). The actin-associated *Myo* gene produces Myo-XI (A) in green algae and gradually extends into ten branches (Peremyslov et al., 2011). The aquaporin-encoding gene *AQP* developed from the *LIPS* type gene in green algae and gradually diverged into eight significantly different *AQP* genes (GIPS, LIPS, HIPS, XIPS, SIPS, PIPS, TIPS, and NIPS) in various plants, including soybean, upland cotton, and oilseed rape (Hussain et al., 2020). The RNA splice component *NSR/RBP* was slightly extended in soybean but contained differences in its conserved motifs (Lucero et al., 2020; Tables 1, 2; Figure 3).

The *SH3P* gene family, associated with cell plate formation, may have originated from the SH3P1-like ancestor of Charophyta and gradually expanded during the transition to mosses and angiosperms (Forero and Cvrckova, 2019). The cellulose synthase superfamily *CesA*, associated with cell wall formation, developed several branches among different species (CSLA and its developed branches CSLC and CESA, CSLB/H and its developed branches CSLF, CSLJ/M, CSLG, and CSLE). Moreover, the different subfamilies exhibit obvious selection for sugar synthesis. For example, certain members of the CSLJ subfamily may mediate (1, 3;1, 4)-β-glucan biosynthesis (Little et al., 2018). The *FT*/*TFLL* gene family, associated with flowering time, developed from *MFT-like* in angiosperms and contains several members (6) (Jin et al., 2021). The *OFP* gene family, associated with fruit shape, may have originated from the ancestors of land plants. Different species have varying numbers of these genes, which have been divided into 11 classes, due to numerous copy-number loss events (Liu et al., 2014). *HAM* gene families associated with tissue formation were generated from bryophytes and exhibit several molecular differences among different plant classes, where each family formed one branch. These gene families expanded in seed plants and ultimately evolved into two angiosperm branches (Type-I and Type-II) (Geng et al., 2021; Tables 1, 2; Figure 3).

### Evolution of signal transduction gene families

Studies on signal transduction-related gene families showed that the number of *PAB* gene families, which are involved in promoting mRNA stability and protein translation, varies significantly among different groups. These gene families are divided into three groups (Class I: PAB1/PAB3/PAB5, Class II: PAB2/PAB4/PAB8, and Class III: PAB6/PAB7); however, their individual evolutionary routes remain unknown (Gallie and Liu, 2014). In seed plants, small peptide signal-related *CEP* gene families may have significantly expanded *via* WGD, especially in the Gramineae and Solanaceae (Ogilvie et al., 2014). The *CNGC* gene family, which act in calcium-gating, are divided into five classes (Groups I, II, III, IVA, and IVB), and the number of members within each class varies considerably (Saand et al., 2015). Auxin response factors are classified into three classes and seven groups (Class A: ARF5/7, ARF6/8; Class B: ARF1, ARF2, ARF3/4, ARF9; and Class C: ARF10/16/17) and were formed through the evolution of three bryophyte proteins (Finet et al., 2013). The alkalization factor *RALF* genes are divided into ten classes and may have developed from two primitive ancestors (Cao and Shi, 2012; Tables 1, 2; Figure 3).

The number of *CBL*, *CIPK*, *CDPK*, and *CRK* gene members associated with calcium signaling differs significantly across evolutionary stages (during the transition from lower plants to core angiosperms), and this phenomenon may be due to the abundant occurrence of WGD events and gene loss at these evolutionary stages. These polyploidy events then promoted the functional differentiation of corresponding proteins (Xiao et al., 2017; Zhang X. X. et al., 2020). Although only two *PEBP* genes, which are bind phospholipids and have roles in signal transduction, have been characterized in gymnosperms, they are particularly abundant in angiosperms, and their secondary expansion appears to be related to the formation of seed plants and angiosperms (Hedman et al., 2009; Karlgren et al., 2011). *GPAT* genes, which are associated with glycerol 3-phosphate biosynthesis, emerged earlier than those present in green algae, from which GPAT and GPAT9 developed into several *GPAT* genes in land plants (Waschburger et al., 2018; Tables 1, 2; Figure 3).

### Evolution of other gene families

During evolution, other plant gene families have generated a high number of members with functional differentiation. In the salt or nutrient signaling pathways, the phosphorus transporter-encoding gene (*PHO*) contains obvious differences in copy number [from 0/1 when developed in green algae to two gradually more complex branches (C-1 and C-2) in land plants], protein structure, and number of introns (He et al., 2013). The ion transduction *VP* gene is divided into two branches, II and I, which originated from red algae and green algae, respectively. These branches were affected by polyploidy and were expanded in angiosperms (Zhang Y. M. et al., 2020). The plant ferritin *Fer* gene was already present in red algae and marginally increased in copy number in the later clades. Notably, the *Fer* gene of the monocotyledonous plant *Lycoris aurea* (Asparagales) appears more comparable to that of dicotyledonous plants (Strozycki et al., 2010). *VIT* genes encoding iron transporters consist of five ancient branches; however, two duplication events and six loss events led to substantial contraction of non-angiosperm *VIT* genes, and a subsequent expansion in copy number in angiosperms (Cao, 2019). Meanwhile, there is no significant difference in the number of methionine biosynthesis-related gene family (*CIMS*) members among green plants; however, multiple gene loss and gene duplication events occurred. In addition, WGT (wide-genome triploidy) led to the expansion of *CIMS* genes in soybean and alfalfa (Rody and de Oliveira, 2018; Tables 1, 2; Figure 3).

There has been obvious expansion and gene loss of the β-glucohydrolase (*BAM*) gene in different groups of hydrolases, which were divided into eight branches (Bam1, Bam10, Bam3, Bam4, Bam9, Bam5/6, Bam2/7, and Bam8) that existed before the formation of land plants. However, significant gene losses have occurred in basal land plants (Thalmann et al., 2019). The *SUS* gene family, which is involved in glycolysis, can be divided into three groups containing members that may have developed from WGD and that have also undergone obvious expansion in certain higher plants (Xu et al., 2019). Among the genes related to epigenetic factors, the methylation-related *HMT* family has two branches (Class 1 and Class 2) in land plants, especially in seed plants, indicating that the *HMT* genes underwent two separate functional differentiation events (Zhao et al., 2018). The ubiquitin-related *FBP* family that originated in green algae has undergone significant expansion in lower plants, monocotyledons, and dicotyledons, such as Brassicaceae (Navarro-Quezada et al., 2013; Tables 1, 2; Figure 3).

## Concluding remarks and perspectives

Although it is desirable to develop better plant-based products and improve plant stress resistance for commercial reasons, it can be challenging to decipher the molecular profiles of plants and efficiently generate molecular resources (Nelson and Werck-Reichhart, 2011; Zhang et al., 2019). The development of plant molecular biology techniques has enabled the key events in plant evolution to be systematically characterized, including the molecular mechanisms underlying the adaptation of plants to life on land and plant hybrid formation (Cheng et al., 2019; Wang et al., 2021). To adequately assess the molecular evolution of plants, it is necessary to investigate a large variety of plant gene families. In particular, it is critical to analyze the unique features of the origin and evolutionary branches of different gene families.

The evidence described in this review suggests that gene duplication and gene loss occurred in nearly all gene families during plant evolution. Genes encoding TFs, proteins involved in disease and stress resistance, structural proteins, and signal transduction-related proteins have been extensively studied compared to genes in the hydrolase gene family (Shao et al., 2019; Lucero et al., 2020; Jin et al., 2021). Moreover, most research on molecular evolution has employed a small number of species and lacks systematics analysis. Therefore, it is necessary to conduct large-scale evolutionary studies on a broader selection of species groups, as well as the evolution of other functional genes, such as those encoding RNA-modifying proteins and autophagy-associated proteins.

Considering the content of these related studies, we believe that the following three aspects can be explored in the future to promote the understanding of plant molecular evolution-related processes. (A) the subfunctionalization of large families and the systematic evolutionary patterns of signaling pathways; (B) the comprehensiveness of the selection of representative plant taxa in molecular evolution studies and the statistical determination of related properties; (C) the origin of families, especially gene families associated with specific evolutionary events.

In summary, we have reviewed the molecular evolution of plants and discussed the potential contributions, challenges, and strategies associated with the gene families involved in the molecular evolution of plants as plants adapted to terrestrial environments and developed resistance to stress. The formation of different plant taxonomic units is closely associated with various plant gene families and their subsequent changes, most of which are characterized by traits that promote their environmental adaptability (Cheng et al., 2019; Shao et al., 2019; Man et al., 2020; Schilling et al., 2020). The transition of basal plants, such as Spiragloeophycidae and Streptophyte algae, often involved elaborate mechanisms to enhance plant resistance to environmental stress. For example, differences in the degree of water dependence and oxygen use occurred during the adaptation of plants for terrestrial environments. Investigation into relevant molecules, such as proteins encoded by key genes associated with the plant transition to terrestrial environments, can provide a pathway to enhancing the natural resistance of plants, thereby reducing their dependence on environmental growth conditions, and improving crop yield (Cheng et al., 2019; Figure 3).

## Author contributions

YF wrote the manuscript. XL, JJ, XH, JG, DZ, and XX completed the revision of the manuscript. All authors contributed to the article and approved the submitted version.

## References

[B1] Airoldi C. A. Davies B. (2012). Gene duplication and the evolution of plant MADS-box transcription factors. *J. Genet. Genom.* 39 157–165. 10.1016/j.jgg.2012.02.008 22546537

[B2] Bondino H. G. Valle E. M. Ten Have A. (2012). Evolution and functional diversification of the small heat shock protein/alpha-crystallin family in higher plants. *Planta* 235 1299–1313. 10.1007/s00425-011-1575-9 22210597

[B3] Borrelli G. M. Mazzucotelli E. Marone D. Crosatti C. Michelotti V. Vale G. (2018). Regulation and evolution of NLR genes: A close interconnection for plant immunity. *Int. J. Mol. Sci.* 19:1662. 10.3390/ijms19061662 29867062 PMC6032283

[B4] Boscolo-Galazzo F. Crichton K. A. Ridgwell A. Mawbey E. M. Wade B. S. Pearson P. N. (2021). Temperature controls carbon cycling and biological evolution in the ocean twilight zone. *Science* 371 1148–1152. 10.1126/science.abb6643 33707262

[B5] Cao J. (2016). Analysis of the Prefoldin gene family in 14 plant species. *Front. Plant Sci.* 7:317. 10.3389/fpls.2016.00317 27014333 PMC4792155

[B6] Cao J. (2019). Molecular evolution of the vacuolar iron transporter (VIT) family genes in 14 plant species. *Genes* 10:144. 10.3390/genes10020144 30769903 PMC6409731

[B7] Cao J. Shi F. (2012). Evolution of the RALF gene family in plants: Gene duplication and selection patterns. *Evol. Bioinform.* 8 271–292. 10.4137/EBO.S9652 22745530 PMC3382376

[B8] Cao J. Li X. Y. Lv Y. Q. (2017). Dynein light chain family genes in 15 plant species: Identification, evolution and expression profiles. *Plant Sci.* 254 70–81. 10.1016/j.plantsci.2016.10.011 27964786

[B9] Cao J. Lv Y. Q. Hou Z. R. Li X. Ding L. N. (2016). Expansion and evolution of thaumatin-like protein (TLP) gene family in six plants. *Plant Growth Regul.* 79 299–307. 10.1007/s10725-015-0134-y

[B10] Cheng S. Xian W. Fu Y. Marin B. Keller J. Wu T. (2019). Genomes of subaerial Zygnematophyceae provide insights into land plant evolution. *Cell* 179 1057–1067. 10.1016/j.cell.2019.10.019 31730849

[B11] Clavel M. Pelissier T. Montavon T. Tschopp M. A. Pouch-Pelissier M. N. Descombin J. (2016). Evolutionary history of double-stranded RNA binding proteins in plants: Identification of new cofactors involved in easiRNA biogenesis. *Plant Mol. Biol.* 91 131–147. 10.1007/s11103-016-0448-9 26858002

[B12] Dong S. S. Liu M. Liu Y. Chen F. Yang T. Chen L. (2021). The genome of Magnolia biondii Pamp. Provides insights into the evolution of Magnoliales and biosynthesis of terpenoids. *Hortic. Res.* 8:38. 10.1038/s41438-021-00471-9 33642574 PMC7917104

[B13] Duplais C. Papon N. Courdavault V. (2020). Tracking the origin and evolution of plant metabolites. *Trends Plant Sci.* 25 1182–1184. 10.1016/j.tplants.2020.08.010 32896488

[B14] Feng G. Q. Burleigh J. G. Braun E. L. Mei W. B. Barbazuk W. B. (2017). Evolution of the 3R-MYB gene family in plants. *Genome Biol. Evol.* 9 1013–1029. 10.1093/gbe/evx056 28444194 PMC5405339

[B15] Finet C. Berne-Dedieu A. Scutt C. P. Marletaz F. (2013). Evolution of the ARF gene family in land plants: Old domains, new tricks. *Mol. Biol. Evol.* 30 45–56. 10.1093/molbev/mss220 22977118

[B16] Finet C. Floyd S. K. Conway S. J. Zhong B. J. Scutt C. P. Bowmanb J. L. (2016). Evolution of the YABBY gene family in seed plants. *Evol. Dev.* 18 116–126. 10.1111/ede.12173 26763689

[B17] Forero A. B. Cvrckova F. (2019). SH3Ps-evolution and diversity of a family of proteins engaged in plant cytokinesis. *Int. J. Mol. Sci.* 20:5623. 10.3390/ijms20225623 31717902 PMC6888108

[B18] Gallie D. R. Liu R. Y. (2014). Phylogenetic analysis reveals dynamic evolution of the poly(A)-binding protein gene family in plants. *BMC Evol. Biol.* 14:238. 10.1186/s12862-014-0238-4 25421536 PMC4252990

[B19] Gao B. Chen M. X. Li X. S. Liang Y. Q. Zhang D. Y. Wood A. J. (2020). Ancestral gene duplications in mosses characterized by integrated phylogenomic analyses. *J. Syst. Evol.* 60 144–159. 10.1111/jse.12683

[B20] Geng Y. Guo L. Han H. Liu X. Banks J. A. Wisecaver J. H. (2021). Conservation and diversification of HAIRY MERISTEM gene family in land plants. *Plant J.* 106 366–378. 10.1111/tpj.15169 33484592

[B21] Gramzow L. Ritz M. S. Theissen G. (2010). On the origin of MADS-domain transcription factors. *Trends Genet.* 26 149–153. 10.1016/j.tig.2010.01.004 20219261

[B22] Guo H. S. Zhang Y. M. Sun X. Q. Li M. M. Hang Y. Y. Xue J. Y. (2016). Evolution of the KCS gene family in plants: The history of gene duplication, sub/neofunctionalization and redundancy. *Mol. Genet. Genom.* 291 739–752. 10.1007/s00438-015-1142-3 26563433

[B23] He L. L. Zhao M. Wang Y. Gai J. Y. He C. Y. (2013). Phylogeny, structural evolution and functional diversification of the plant PHOSPHATE1 gene family: A focus on Glycine max. *BMC Evol. Biol.* 13:103. 10.1186/1471-2148-13-103 23705930 PMC3680083

[B24] Hedman H. Kallman T. Lagercrantz U. (2009). Early evolution of the MFT-like gene family in plants. *Plant Mol. Biol.* 70 359–369. 19288213 10.1007/s11103-009-9478-x

[B25] Hsu H. F. Chen W. H. Shen Y. H. Hsu W. H. Mao W. T. Yang C. H. (2021). Multifunctional evolution of B and AGL6 MADS box genes in orchids. *Nat. Commun.* 12:902. 10.1038/s41467-021-21229-w 33568671 PMC7876132

[B26] Hussain A. Tanveer R. Mustafa G. Farooq M. Amin I. Mansoor S. (2020). Comparative phylogenetic analysis of aquaporins provides insight into the gene family expansion and evolution in plants and their role in drought tolerant and susceptible chickpea cultivars. *Genomics* 112 263–275. 10.1016/j.ygeno.2019.02.005 30826442

[B27] Ifuku K. Ishihara S. Shimamoto R. Ido K. Sato F. (2008). Structure, function, and evolution of the PsbP protein family in higher plants. *Photosynth. Res.* 98 427–437. 10.1007/s11120-008-9359-1 18791807

[B28] Jin J. Tian F. Yang D. C. Meng Y. Q. Kong L. Luo J. (2017). PlantTFDB 4.0: Toward a central hub for transcription factors and regulatory interactions in plants. *Nucleic Acids Res.* 45 D1040–D1045. 10.1093/nar/gkw982 27924042 PMC5210657

[B29] Jin S. Nasim Z. Susila H. Ahn J. H. (2021). Evolution and functional diversification of flowering locus T/terminal flower 1 family genes in plants. *Semin. Cell Dev. Biol.* 109 20–30. 10.1016/j.semcdb.2020.05.007 32507412

[B30] Karlgren A. Gyllenstrand N. Kallman T. Sundstrom J. F. Moore D. Lascoux M. (2011). Evolution of the PEBP gene family in plants: Functional diversification in seed plant evolution. *Plant Physiol.* 156 1967–1977. 10.1104/pp.111.176206 21642442 PMC3149940

[B31] Lafon-Placette C. Vallejo-Marin M. Parisod C. Abbott R. J. Kohler C. (2016). Current plant speciation research: Unravelling the processes and mechanisms behind the evolution of reproductive isolation barriers. *New Phytol.* 209 29–33. 10.1111/nph.13756 26625345

[B32] Li J. Yang S. Yang X. Wu H. Tang H. Yang L. (2022). PlantGF: An analysis and annotation platform for plant gene families. *Database* 2022:baab088. 10.1093/database/baab088 35134149 PMC9278324

[B33] Li L. Z. Wang S. B. Wang H. L. Sahu S. K. Marin B. Li H. Y. (2020). The genome of *Prasinoderma coloniale* unveils the existence of a third phylum within green plants. *Nat. Ecol. Evol.* 4:1220. 10.1038/s41559-020-1221-7 32572216 PMC7455551

[B34] Li W. Y. Liu B. Yu L. J. Feng D. R. Wang H. B. Wang J. F. (2009). Phylogenetic analysis, structural evolution and functional divergence of the 12-oxo-phytodienoate acid reductase gene family in plants. *BMC Evol. Biol.* 9:90. 10.1186/1471-2148-9-90 19416520 PMC2688005

[B35] Li W. Liu W. Wei H. L. He Q. L. Chen J. H. Zhang B. H. (2014). Species-specific expansion and molecular evolution of the 3-hydroxy-3-methylglutaryl coenzyme a reductase (HMGR) gene family in plants. *PLoS One* 9:e94172. 10.1371/journal.pone.0094172 24722776 PMC3983158

[B36] Lian G. B. Ding Z. W. Wang Q. Zhang D. B. Xu J. (2014). Origins and evolution of wuschel-related homeobox protein family in plant kingdom. *Sci. World J.* 2017:534140. 10.1155/2014/534140 24511289 PMC3913392

[B37] Liang Z. Geng Y. K. Ji C. M. Du H. Wong C. E. Zhang Q. (2020). *Mesostigma viride* genome and transcriptome provide insights into the origin and evolution of Streptophyta. *Adv. Sci.* 7:1901850. 10.1002/advs.201901850 31921561 PMC6947507

[B38] Little A. Schwerdt J. G. Shirley N. J. Khor S. F. Neumann K. O’Donovan L. A. (2018). Revised phylogeny of the cellulose synthase gene superfamily: Insights into cell wall evolution. *Plant Physiol.* 177 1124–1141. 10.1104/pp.17.01718 29780036 PMC6052982

[B39] Liu D. Sun W. Yuan Y. W. Zhang N. Hayward A. Liu Y. L. (2014). Phylogenetic analyses provide the first insights into the evolution of OVATE family proteins in land plants. *Ann. Bot.* 113 1219–1233. 10.1093/aob/mcu061 24812252 PMC4030818

[B40] Liu M. Wang M. Yang J. Wen J. Guo P. Wu Y. (2019). Evolutionary and comparative expression analyses of TCP transcription factor gene family in land plants. *Int. J. Mol. Sci.* 20:3591. 10.3390/ijms20143591 31340456 PMC6679135

[B41] Lucero L. Bazin J. Melo J. R. Ibanez F. Crespi M. D. Ariel F. (2020). Evolution of the small family of alternative splicing modulators nuclear speckle rna-binding proteins in plants. *Genes* 11:207. 10.3390/genes11020207 32085457 PMC7073835

[B42] Man J. R. Gallagher J. P. Bartlett M. (2020). Structural evolution drives diversification of the large LRR-RLK gene family. *New Phytol.* 226 1492–1505. 10.1111/nph.16455 31990988 PMC7318236

[B43] Mukherjee K. Campos H. Kolaczkowski B. (2013). Evolution of animal and plant dicers: Early parallel duplications and recurrent adaptation of antiviral RNA binding in plants. *Mol. Biol. Evol.* 30 627–641. 10.1093/molbev/mss263 23180579 PMC3563972

[B44] Naramoto S. Hata Y. Kyozuka J. (2020). The origin and evolution of the ALOG proteins, members of a plant-specific transcription factor family, in land plants. *J. Plant Res.* 133 323–329. 10.1007/s10265-020-01171-6 32052256

[B45] Navarro-Quezada A. Schumann N. Quint M. (2013). Plant F-Box protein evolution is determined by lineage-specific timing of major gene family expansion waves. *PLoS One* 8:e68672. 10.1371/journal.pone.0068672 23904908 PMC3719486

[B46] Nelson D. Werck-Reichhart D. (2011). A P450-centric view of plant evolution. *Plant J.* 66 194–211. 10.1111/j.1365-313X.2011.04529.x 21443632

[B47] Ng M. Yanofsky M. F. (2001). Function and evolution of the plant MADS-box gene family. *Nat. Rev. Genet.* 2 186–195. 10.1038/35056041 11256070

[B48] Nikolov L. A. Runions A. Das Gupta M. Tsiantis M. (2019). Leaf development and evolution. *Curr. Top. Dev. Biol.* 131:109. 10.1016/bs.ctdb.2018.11.006 30612614

[B49] Ogilvie H. A. Imin N. Djordjevic M. A. (2014). Diversification of the C-Terminally Encoded Peptide (CEP) gene family in angiosperms, and evolution of plant-family specific CEP genes. *BMC Genom.* 15:870. 10.1186/1471-2164-15-870 25287121 PMC4197245

[B50] Peremyslov V. Mockler T. C. Filichkin S. A. Fox S. E. Jaiswal P. Makarova K. S. (2011). Expression, splicing, and evolution of the myosin gene family in plants. *Plant Physiol.* 15 1191–1204. 10.1104/pp.110.170720 21233331 PMC3046578

[B51] Preston J. C. Hileman L. C. (2013). Functional evolution in the plant Squamosa-Promoter Binding Protein-Like (SPL) gene family. *Front. Plant Sci.* 4:80. 10.3389/fpls.2013.00080 23577017 PMC3617394

[B52] Pu X. J. Lv X. Lin H. H. (2015). Unraveling the evolution and regulation of the alternative oxidase gene family in plants. *Dev. Genes Evol.* 225 331–339. 10.1007/s00427-015-0515-2 26438244

[B53] Qiu Y. Claudia K. (2021). Endosperm evolution by duplicated and neofunctionalized type I MADS-box transcription factors. *Mol. Biol. Evol.* 39:msab355. 10.1093/molbev/msab355 34897514 PMC8788222

[B54] Reeves P. A. Olmstead R. G. (2003). Evolution of the TCP gene family in Asteridae: Csladistic and network approaches to understanding regulatory gene family diversification and its impact on morphological evolution. *Mol. Biol. Evol.* 20 1997–2009. 10.1093/molbev/msg211 12885953

[B55] Rody H. V. S. de Oliveira L. O. (2018). Evolutionary history of the cobalamin-independent methionine synthase gene family across the land plants. *Mol. Phylogenet. Evol.* 120 33–42. 10.1016/j.ympev.2017.12.003 29222062

[B56] Saand M. A. Xu Y. P. Munyampundu J. P. Li W. Zhang X. R. Cai X. Z. (2015). Phylogeny and evolution of plant cyclic nucleotide-gated ion channel (CNGC) gene family and functional analyses of tomato CNGCs. *DNA Res.* 22 471–483. 10.1093/dnares/dsv029 26546226 PMC4675716

[B57] Schilling S. Kennedy A. Pan S. Jermiin L. S. Melzer R. (2020). Genome-wide analysis of MIKC-type MADS-box genes in wheat: Pervasive duplications, functional conservation and putative neofunctionalization. *New Phytol.* 225 511–529. 10.1111/nph.16122 31418861

[B58] Schneider H. Liu H. M. Chang Y. F. Ohlsen D. Perrie L. R. Shepherd L. (2017). Neo- and Paleopolyploidy contribute to the species diversity of *Asplenium*-the most species-rich genus of ferns. *J. Syst. Evol.* 55 353–364. 10.1111/jse.12271

[B59] Shao Z. Xue J. Wang Q. Wang B. Chen J. (2019). Revisiting the origin of plant NBS-LRR genes. *Trends Plant Sci.* 24 9–12. 10.1016/j.tplants.2018.10.015 30446304

[B60] Singh R. K. Gase K. Baldwin I. T. Pandey S. P. (2015). Molecular evolution and diversification of the Argonaute family of proteins in plants. *BMC Plant Biol.* 15:23. 10.1186/s12870-014-0364-6 25626325 PMC4318128

[B61] Song B. Buckler E. Wang H. Wu Y. Rees E. Kellogg E. (2021). Conserved noncoding sequences provide insights into regulatory sequence and loss of gene expression in maize. *Genome Res.* 31 1245–1257. 10.1101/gr.266528.120 34045362 PMC8256870

[B62] Song X. M. Wang J. P. Sun P. C. Ma X. Yang Q. H. Hu J. J. (2020). Preferential gene retention increases the robustness of cold regulation in Brassicaceae and other plants after polyploidization. *Hortic. Res.* 7:20. 10.1038/s41438-020-0253-0 32133148 PMC7035258

[B63] Strozycki P. M. Szymanski M. Szczurek A. Barciszewski J. Figlerowicz M. (2010). A new family of Ferritin genes from lupinus luteus-comparative analysis of plant ferritins, their gene structure, and evolution. *Mol. Biol. Evol.* 27 91–101. 10.1093/molbev/msp196 19726535

[B64] Su D. Yang L. Shi X. Ma X. Zhou X. Hedges S. B. (2021). Large-scale phylogenomic analyses reveal the monophyly of bryophytes and Neoproterozoic origin of land plants. *Mol. Biol. Evol.* 38 3332–3344. 10.1093/molbev/msab106 33871608 PMC8321542

[B65] Sun W. Ma Z. Liu M. (2021). Plant cytochrome P450 plasticity and evolution. *Mol. Plant* 14 1244–1265. 10.1016/j.molp.2021.06.028 34216829

[B66] Thalmann M. Coiro M. Meier T. Wicker T. Zeeman S. C. Santelia D. (2019). The evolution of functional complexity within the -amylase gene family in land plants. *BMC Evol. Biol.* 19:66. 10.1186/s12862-019-1395-2 30819112 PMC6394054

[B67] Theissen G. Melzer R. Rumpler F. (2016). MADS-domain transcription factors and the floral quartet model of flower development: Linking plant development and evolution. *Development* 143 3259–3271. 10.1242/dev.134080 27624831

[B68] Vasco A. Smalls T. L. Graham S. W. Cooper E. D. Wong G. K. S. Stevenson D. W. (2016). Challenging the paradigms of leaf evolution: Class III HD-Zips in ferns and lycophytes. *New Phytol.* 212 745–758. 10.1111/nph.14075 27385116

[B69] Vilela M. M. Del Bem L. E. Van Sluys M. A. de Setta N. Kitajima J. P. Cruz G. M. (2017). Analysis of three sugarcane homo/homeologous regions suggests independent polyploidization events of *Saccharum officinarum* and *Saccharum spontaneum*. *Genome Biol. Evol.* 9 266–278. 10.1093/gbe/evw293 28082603 PMC5381655

[B70] Wang J. P. Yu J. G. Li J. Sun P. C. Wang L. Yuan J. Q. (2018). Two likely auto-tetraploidization events shaped kiwifruit genome and contributed to establishment of the Actinidiaceae family. *iScience* 7:230. 10.1016/j.isci.2018.08.003 30267683 PMC6161637

[B71] Wang J. Qin J. Sun P. Ma X. Yu J. Li Y. (2019). Polyploidy index and its implications for the evolution of polyploids. *Front. Genet.* 10:807. 10.3389/fgene.2019.00807 31552101 PMC6746930

[B72] Wang X. Feng H. Chang Y. Ma C. Wang L. Hao X. (2020). Population sequencing enhances understanding of tea plant evolution. *Nat. Commun.* 11:4447. 10.1038/s41467-020-18228-8 32895382 PMC7477583

[B73] Wang Y. M. Yang Q. Liu Y. J. Yang H. L. (2016). Molecular evolution and expression divergence of the Aconitase (ACO) gene family in land plants. *Front. Plant Sci.* 7:1879. 10.3389/fpls.2016.01879 28018410 PMC5149538

[B74] Wang Z. Jiang Y. Bi H. Lu Z. Ma Y. Yang X. (2021). Hybrid speciation via inheritance of alternate alleles of parental isolating genes. *Mol. Plant* 14 208–222. 10.1016/j.molp.2020.11.008 33220509

[B75] Waschburger E. Kulcheski F. R. Veto N. M. Margis R. Margis-Pinheiro M. Turchetto-Zolet A. C. (2018). Genome-wide analysis of the glycerol-3-phosphate acyltransferase (GPAT) gene family reveals the evolution and diversification of plant GPATs. *Genet. Mol. Biol.* 41 355–370. 10.1590/1678-4685-Gmb-2017-0076 29583156 PMC5913721

[B76] Wu W. T. Liu Y. X. Wang Y. Q. Li H. M. Liu J. X. Tan J. X. (2017). Evolution analysis of the Aux/IAA gene family in plants shows dual origins and variable nuclear localization signals. *Int. J. Mol. Sci.* 18:2107. 10.3390/ijms18102107 28991190 PMC5666789

[B77] Xiao X. H. Yang M. Sui J. L. Qi J. Y. Fang Y. J. Hu S. N. (2017). The calcium-dependent protein kinase (CDPK) and CDPK-related kinase gene families in *Hevea brasiliensis*-comparison with five other plant species in structure, evolution, and expression. *FEBS Open Bio* 7 4–24. 10.1002/2211-5463.12163 28097084 PMC5221434

[B78] Xu X. Y. Yang Y. H. Liu C. X. Sun Y. M. Zhang T. Hou M. L. (2019). The evolutionary history of the sucrose synthase gene family in higher plants. *BMC Plant Biol.* 19:566. 10.1186/s12870-019-2181-4 31852440 PMC6921546

[B79] Yang Z. F. Gu S. L. Wang X. F. Li W. J. Tang Z. X. Xu C. W. (2008). Molecular evolution of the CPP-like gene family in plants: Insights from comparative genomics of *Arabidopsis* and rice. *J. Mol. Evol.* 67 266–277. 10.1007/s00239-008-9143-z 18696028

[B80] Yu X. Xiao J. Chen S. Yu Y. Ma J. Lin Y. (2020). Metabolite signatures of diverse *Camellia sinensis* tea populations. *Nat. Commun.* 11:5586. 10.1038/s41467-020-19441-1 33149146 PMC7642434

[B81] Zhang K. Wang X. W. Cheng F. (2019). Plant polyploidy: Origin, evolution, and its influence on crop domestication. *Hortic. Plant. J.* 5 231–239. 10.1016/j.hpj.2019.11.003

[B82] Zhang L. S. Chen F. Zhang X. T. Li Z. Zhao Y. Y. Lohaus R. (2020). The water lily genome and the early evolution of flowering plants. *Nature* 577 79–84. 10.1038/s41586-019-1852-5 31853069 PMC7015852

[B83] Zhang X. X. Li X. X. Zhao R. Zhou Y. Jiao Y. N. (2020). Evolutionary strategies drive a balance of the interacting gene products for the CBL and CIPK gene families. *New Phytol.* 226 1506–1516. 10.1111/nph.16445 31967665

[B84] Zhang Y. M. Feng X. Wang L. H. Su Y. P. Chu Z. D. Sun Y. X. (2020). The structure, functional evolution, and evolutionary trajectories of the H+-PPase gene family in plants. *BMC Genom.* 21:195. 10.1186/s12864-020-6604-2 32122295 PMC7053079

[B85] Zhao J. F. Favero D. S. Qiu J. W. Roalson E. H. Neff M. M. (2014). Insights into the evolution and diversification of the AT-hook Motif Nuclear Localized gene family in land plants. *BMC Plant Biol.* 14:266. 10.1186/s12870-014-0266-7 25311531 PMC4209074

[B86] Zhao M. Chen P. Wang W. Y. Yuan F. J. Zhu D. H. Wang Z. (2018). Molecular evolution and expression divergence of HMT gene family in plants. *Int. J. Mol. Sci.* 19:1248. 10.3390/ijms19041248 29677135 PMC5979542

[B87] Zong J. Yao X. Yin J. Zhang D. Ma H. (2009). Evolution of the RNA-dependent RNA polymerase (RdRP) genes: Duplications and possible losses before and after the divergence of major eukaryotic groups. *Gene* 447 29–39. 10.1016/j.gene.2009.07.004 19616606

